# Novel Kidins220/ARMS Splice Isoforms: Potential Specific Regulators of Neuronal and Cardiovascular Development

**DOI:** 10.1371/journal.pone.0129944

**Published:** 2015-06-17

**Authors:** Nathalie Schmieg, Claire Thomas, Arisa Yabe, David S. Lynch, Teresa Iglesias, Probir Chakravarty, Giampietro Schiavo

**Affiliations:** 1 Molecular Neuropathobiology Laboratory, Sobell Department of Motor Neuroscience & Movement Disorders, UCL Institute of Neurology, University College London, London WC1N 3BG, United Kingdom; 2 The Francis Crick Institute, 44 Lincoln’s Inn Fields, London WC2A 3LY, United Kingdom; 3 Leonard Wolfson Centre for Experimental Neurology, University College London, 8 Queen Anne Street, London W1G 9LD, United Kingdom; 4 Instituto de Investigaciones Biomédicas "Alberto Sols" (CSIC-UAM), C/ Arturo Duperier, 4, Madrid 28029, Spain; 5 CIBERNED (ISCIII), C/ Valderrebollo 5, Madrid 28031, Spain; University of Queensland, AUSTRALIA

## Abstract

Kidins220/ARMS is a transmembrane protein playing a crucial role in neuronal and cardiovascular development. Kidins220/ARMS is a downstream target of neurotrophin receptors and interacts with several signalling and trafficking factors. Through computational modelling, we found two potential sites for alternative splicing of Kidins220/ARMS. The first is located between exon 24 and exon 29, while the second site replaces exon 32 by a short alternative terminal exon 33. Here we describe the conserved occurrence of several Kidins220/ARMS splice isoforms at RNA and protein levels. Kidins220/ARMS splice isoforms display spatio-temporal regulation during development with distinct patterns in different neuronal populations. Neurotrophin receptor stimulation in cortical and hippocampal neurons and neuroendocrine cells induces specific Kidins220/ARMS splice isoforms and alters the appearance kinetics of the full-length transcript. Remarkably, alternative terminal exon splicing generates Kidins220/ARMS variants with distinct cellular localisation: Kidins220/ARMS containing exon 32 is targeted to the plasma membrane and neurite tips, whereas Kidins220/ARMS without exon 33 mainly clusters the full-length protein in a perinuclear intracellular compartment in PC12 cells and primary neurons, leading to a change in neurotrophin receptor expression. Overall, this study demonstrates the existence of novel Kidins220/ARMS splice isoforms with unique properties, revealing additional complexity in the functional regulation of neurotrophin receptors, and potentially other signalling pathways involved in neuronal and cardiovascular development.

## Introduction

The development of a fertilised egg into an adult organism requires massive changes in gene expression in a relatively short amount of time. To overcome these challenges, many genes undergo alternative pre-mRNA splicing to regulate gene expression and to generate spatio-temporally encoded protein isoforms with different functions. Up to two thirds of human genes are estimated to undergo alternative splicing throughout development, and impairments of certain splicing events have been linked to genetic diseases [1–4]. For example, the specific expression patterns of alternative splice isoforms of GPR56 play a role in folding and shaping of gyri in mammalian forebrains, while an in frame 15 bp deletion mutation in GPR56 leads to the selective bilateral disruption of the Sylvian fissure [5].

During embryogenesis, the development of many tissues, including the nervous and cardiovascular systems, relies on the correct distribution of growth factors and their receptors, which is finely tuned by alternative splicing at the level of their mRNA or interacting partners [2, 6–10]. Members of the neurotrophin (NT) family and their receptors tropomyosin receptor kinases TrkA, TrkB, TrkC and p75^NTR^ are major players in the development of the nervous system. Upon ligand binding, Trk receptors promote neuronal growth and survival, whereas p75^NTR^ modulates NT signalling via its heterodimerisation with Trk receptors [11]. Additionally, p75^NTR^ binds to pro-neurotrophins to initiate separate signalling cascades that antagonise the effects of NT, indicating that the balance between pro- and mature NT plays a critical role in fine-tuning the signalling output of these growth factors [12].

Alternative splicing provides an additional layer of regulation to these events. Brain-derived neurotrophic factor (BDNF), a TrkB ligand, undergoes alternative splicing [13, 14], which finely regulates mRNA localisation and as result its expression at distinct locations in axon and dendrites [15, 16]. Furthermore, mRNA levels of specific splice variants of BDNF have been found to be altered in Alzheimer’s disease (AD) patients [17]. TrkB itself has various splice isoforms, which have been shown to differentially regulate TrkB signalling [18]. Interestingly, the expression of a truncated Trk splice variant is specifically enhanced by amyloid beta (Aß) and may function as a compensatory response in AD neurons to promote cell survival [19]. A similar compensatory mechanism is likely to occur in motor neurons in which the intracellular sorting and trafficking of TrkB receptors is impaired by down-regulation of the dynein adaptor BICD1 [20].

In addition to the nervous system, NTs and their receptors are essential factors in the formation of the heart and critical regulators of vascular development. NTs modulate angiogenesis and vasculogenesis, operating in close synergy with vascular endothelial growth factor (VEGF), its receptors (VEGFRs) and co-receptor neuropilin 1 (Nrp1) [21]. Although nervous and cardiovascular systems were considered to undergo independent developmental programs, recent evidence suggests that tight links exist between them. However, a precise understanding of the mechanisms enabling functional crosstalk between these tissues remains speculative [22].

Kinase D Interacting Substrate of 220 kDa (Kidins220), also known as Ankyrin-repeat Rich Membrane Spanning protein (ARMS), has been proposed as a signalling/trafficking mediator between these systems [23]. Indeed, mice lacking Kidins220 display striking developmental abnormalities in heart, vasculature and nervous system [24, 25].

Kidins220 binds to Trk receptors via its fourth transmembrane domain [26] and to p75^NTR^ at the endmost 250 carboxy-terminal residues. Kidins220 has been demonstrated to form a ternary complex with Trk receptors and p75^NTR^, altering the affinity of nerve growth factor (NGF) for TrkA [27]. Stimulation with NGF or BDNF induces the phosphorylation of Kidins220, suggesting that Kidins220 may be a direct substrate for these receptor tyrosine kinases [28]. Based on these findings, it was suggested that Kidins220 might be involved in fine-tuning the affinity of NT for their receptors [27]. Interestingly, NT receptors are not the only receptor tyrosine kinases that interact with Kidins220. In fact, Kidins220 has been shown to bind to VEGFR-2 and 3 [25], and its genetic ablation in mice generates vascular phenotypes closely resembling those observed in Nrp1 knockout animals [29, 30]. These findings lead to the hypothesis that Kidins220 plays a crucial role in modulating the crosstalk between different signalling pathways [23].

The carboxy-terminal region of Kidins220 hosts a kinesin-interacting motif (KIM), which binds to kinesin light chain 1 and 2, and recruit the kinesin-1 motor complex. Kidins220 mutants lacking a functional KIM domain display impaired neurite transport in PC12 cells and deficits in NT signalling and neurite differentiation [31]. Kidins220 might therefore link signalling receptors to motor proteins and regulate the intracellular trafficking of receptor complexes to their target destinations [23].

Recently, a further connection to the intracellular trafficking machinery has been identified. The carboxy-terminal region of Kidins220 and in particular its PDZ binding motif interacts with SNX27, a member of the sorting nexin family [32]. SNX27 is a cargo specific adaptor of the retromer complex [33], which regulates multiple cargo-sorting events within the endosomal network. Knockdown of SNX27 causes a strong decrease in the plasma membrane levels of Kidins220 in HeLa cells [32]. Moreover, knockdown of Vps35, which is responsible for cargo recognition within the retromer complex, also decreases the cell surface expression of Kidins220 [32].

The p75^NTR^ binding site, the KIM domain and the PDZ binding domain are located at the carboxy-terminus of Kidins220 and are all encoded in mouse by exon 32. This work provides the first functional characterisation of the alternative splicing variants of Kidins220, including two alternative terminal exon (ATE) splice forms, which lead to changes of Kidins220’s carboxy-terminus. Using rat PC12 cells, mouse primary neurons, and mouse and human tissue samples, we provide evidence that Kidins220 undergoes a finely-tuned spatio-temporal regulation of splicing in mouse and human tissues, which is likely to be conserved also in other species. Interestingly, the expression of specific Kidins220 splice isoforms is regulated by NT, suggesting the existence of a feedback mechanism linking NT signalling and the expression of scaffolding proteins controlling NT receptor complex assembly and trafficking.

## Materials and Methods

### Ethics statement

All experiments were carried out following the guidelines of the Cancer Research UK genetic manipulation and Ethic Committees and in accordance with the European Community Council Directive of November 24, 1986 (86/609/EEC). Animal work was carried out under license from the UK Home Office in accordance with the Animals (Scientific Procedures) Act 1986, which aims to minimise the use of animals and their suffering. When required, mice were sacrificed using the most appropriate Schedule 1 method.

### Reagents

All chemicals and primers were from Sigma, unless stated otherwise. Total RNAs from a panel of human tissues were purchased from Ambion. Each sample represents a pool of healthy tissue from three adults.

### Primers and bioinformatic analysis

Mouse: GCTGCTGAACAGGGCAATGTG (3f); CAGTGCAGTCATCGAATTAGCTCC (8rev); CCCTCCTCGGCCTCCTTCT (24f); CAATACCCGGCCATTTATGT (30 rev); AGCTTTGAAGAACTGAACACG (31f); CAGTCACAAACTCGCAGAACC (31/32f); TTCTAACTGGGACATCTGAGC (32 rev); TTAAACTGGATCTTCTGAACCG (33 rev).

Human: GGCGCTGTCAGAGGTGGTCAT (9f); CATATCCAAGCATGTCACCGTC (13 rev); CAGTATTGGAGGACTGGCGTA (24f); ACTGAGCTAACACACGGCCAT (30rev); TAATATGGATCCGTACTAGAAATGAGAAAC (31f); CTTCTTCAGTGATAGGATCCA (32rev); GCTATCTATCTCAAGGCCAGC (33 rev).

The UCSC (www.genome.ucsc.edu) database was searched for Kidins220 cDNA and expressed sequence tags (ESTs) using full-length mouse Kidins220 (NM_001081378). Mouse, human and rat Kidins220 protein sequences were aligned to the Mouse mm9 genome using tblastn, to determine the exon-intron structure.

The sequences of the new Kidins220 alternative splice isoforms have been submitted to the NCBI database (GenBank) and are published under the accession numbers provided in S1 Table. Sequences of Kidins220 ATEs are shown in S2 Table.

### Constructs

Kidins220 isoforms m1, m6, m1/C2 and m6/C2 were cloned from IMAGE clones accession numbers 2192152, 30543572 and 5363780 (obtained from MRC Geneservice, Cambridge, UK) and RT-PCR of mouse brain tissue. All constructs were expressed in a HA-tagged modified pLVX-Tight-Puro Vector using a Tet-ON pLVX system (Clontech) using manufacturer’s instructions.

The carboxy terminal region of Kidins220 (KC; exons 29–32; amino acid 1209–1762) was inserted in frame in the pGEX-KG vector [31], whereas the alternative carboxy-terminus encoded by exon 33 was inserted into the pGEX-4T3 vector (both from Amersham Biosciences) using BamHI/EcoRI restriction sites. GST fusion proteins were expressed at 30°C for 5 h in *E*. *coli* BL21 bacteria [31] and purified using glutathione-agarose beads (Sigma).

### Antibodies

Polyclonal antibodies against Kidins220 isoform m1 (peptide sequence: NH_2_-EVIKEDAAEGLPSPTASSREKSWTRKQLMELC-CONH_2_) and m6 (peptide sequence: NH_2_-GLSGPQHPFYNRASVPATGTSLLLSSMC-CONH_2_) were raised in rabbit (Pierce-Thermo Fisher). The polyclonal antibody against Exon 32 was described previously [34]. The monoclonal antibody against Kidins220 isoform C2 was raised by BioGenes using purified GST-exon 33 fusion protein. The monoclonal anti-HA (clone 12CA5) was produced by Cancer Research UK using the peptide sequence CENAAPVLDRQRFRRSSLH. The rat anti-HA antibody used in HEK cells was from Roche. The panTrk antibody used for TrkA staining in PC12 cells was obtained from Santa Cruz (C-14).

### RNA/DNA preparation

Total RNAs from a panel of mouse tissues were obtained from an adult C57BL/6Jax mouse. Tissues were homogenised and the RNA extracted using an RNeasy kit (Quiagen) following the manufacturer’s instructions. 2 μg of RNA/sample were reverse transcribed using SuperScript VILO cDNA synthesis kit (Invitrogen) and the resulting cDNA was amplified by either Megamix-Blue (Microzone Ltd) containing recombinant *Taq* polymerase or KOD Hot Start DNA Polymerase (Novagen). Amplified cDNA was run on a 1.5% agarose gel and isolated DNA bands were extracted using a QIAquick Gel Extraction kit (Qiagen) according to manufacturer’s instructions. DNA was amplified using BigDye Terminator Cycle Sequencing Kit (Applied Biosystems) and purified using the DyeEx 2.0 Spin Kit (QIAGEN) prior to sequencing.

### Cell culture

Cortical and hippocampal primary neurons were isolated from embryonic day 18.5 (E18.5) C57BL/6Jax embryos. Cortices or hippocampi were collected in ice-cold phosphate-buffered saline (PBS) and incubated with 0.125% trypsin for 30 or 15 min at 37°C, respectively. Cells were then dissociated mechanically and plated in poly-L-lysine/laminin coated P90 dishes containing plating medium (Dulbecco’s Modified Eagle Medium (DMEM), 10% heat-inactivated horse serum and 2 mM glutamine). 3 h later, the plating medium was replaced by differentiation medium (Neurobasal (Invitrogen), 2% B27 supplement (Gibco), 2 mM glutamine and penicillin/streptomycin).

Motor neurons were isolated from E13.5 C57BL/6Jax embryos [35]. Meninges were removed from isolated spinal cords, and motor neurons were collected in ice cold PBS. Cells were then incubated with 0.025% trypsin in PBS for 10 min at 37°C and disaggregated using L-15 medium (Gibco), 4% bovine serum albumin (BSA) in PBS and DNAse (1 mg/ml). Motor neurons were plated in polyornithine/laminin coated P60 dishes containing motor neuron medium (Neurobasal (Invitrogen), 2% B27 supplement (Gibco), 2% heat inactivated horse serum, 0.5 mM L-glutamine, 25 μM β-mercaptoethanol, 10 ng/ml rat ciliary neurotrophic factor, 100 pg/ml rat glial-derived neurotrophic factor (both R&D Systems) and penicillin/streptomycin).

PC12 cells were provided by Dr. T.R. Rogers (University of Maryland School of Medicine). These cells were grown in DMEM containing 7.5% foetal bovine serum and 7.5% horse serum, 4 mM glutamine and penicillin/streptavidin. PC12 cells were transfected using a Polyplus JetPEI transfection kit (using 0.5 μg pLVX Tet-ON vector and 0.5 μg of the respective Kidins220 isoform construct) and were differentiated with 100 ng/ml NGF (Alomone) for 48 h.

HEK cells stably transfected with full-length TrkA [36] were grown in DMEM containing 10% foetal bovine serum and 4 mM glutamine at 37°C. One day prior to transfection, cells were plated in tetracyclin free medium. For transfection, HEK-TrkA cells were plated on polyornithine-coated coverslips and transfected with Tet-ON pLVX constructs using Polyplus JetPEI as described above. Doxycycline was used to switch on expression 4 h after transfection.

### Immunofluorescence

Transfected PC12 and HEK-TrkA cells were fixed for 15 min with 4% paraformaldehyde in PBS. Cells were washed with PBS, blocked in 2% BSA in PBS containing 0.1% Triton X-100. Cells were tested for Kidins220 splice isoform expression using a mouse monoclonal anti-HA antibody (1:500; 12CA5, Cancer Research UK London Research Institute) and for TrkA expression using a rabbit polyclonal pan-Trk antibody (1:500; C14, Santa Cruz) for 1 h at room temperature. Cells were then washed and incubated with secondary antibodies (1:400 in 2% bovine serum albumin; AlexaFluor555 goat anti-mouse and AlexaFluor488 goat anti-rabbit, Invitrogen) for 30 min at room temperature. Mowiol was used to mount the coverslips. Coverslips were imaged using an LSM510 or LSM780 confocal microscopes (Zeiss) using a 63x Plan Apochromat oil-immersion objective.

## Results

### Computational evidence supports alternative splicing of mouse and human Kidins220

Based on previous results suggesting sequence heterogeneity in the central domain of rat Kidins220 [34], we investigated whether Kidins220 might undergo alternative splicing in this region, which in turn may support its complex functions in the nervous and cardiovascular systems. To examine the exon-intron structure of mouse (S1 Fig) and human (S2 Fig) Kidins220, we searched the UCSC genome database for Kidins220 cDNA and expressed sequence tags (ESTs). We found evidence of potential alternative splicing between exons 24 and 29 of the Kidins220 sequence. We also found evidence for a possible alternative terminal exon (ATE) splicing of Kidins220 by a splice out of the terminal exon 32 and replacement by a much shorter exon 33. These findings appear to be conserved, since we found alternative splicing sites in mouse, rat and human sequences (S3 Fig).

### Alternative splice isoforms of Kidins220 lacking exons 24–29 are present in adult mouse and human tissues

**Fig 1:**
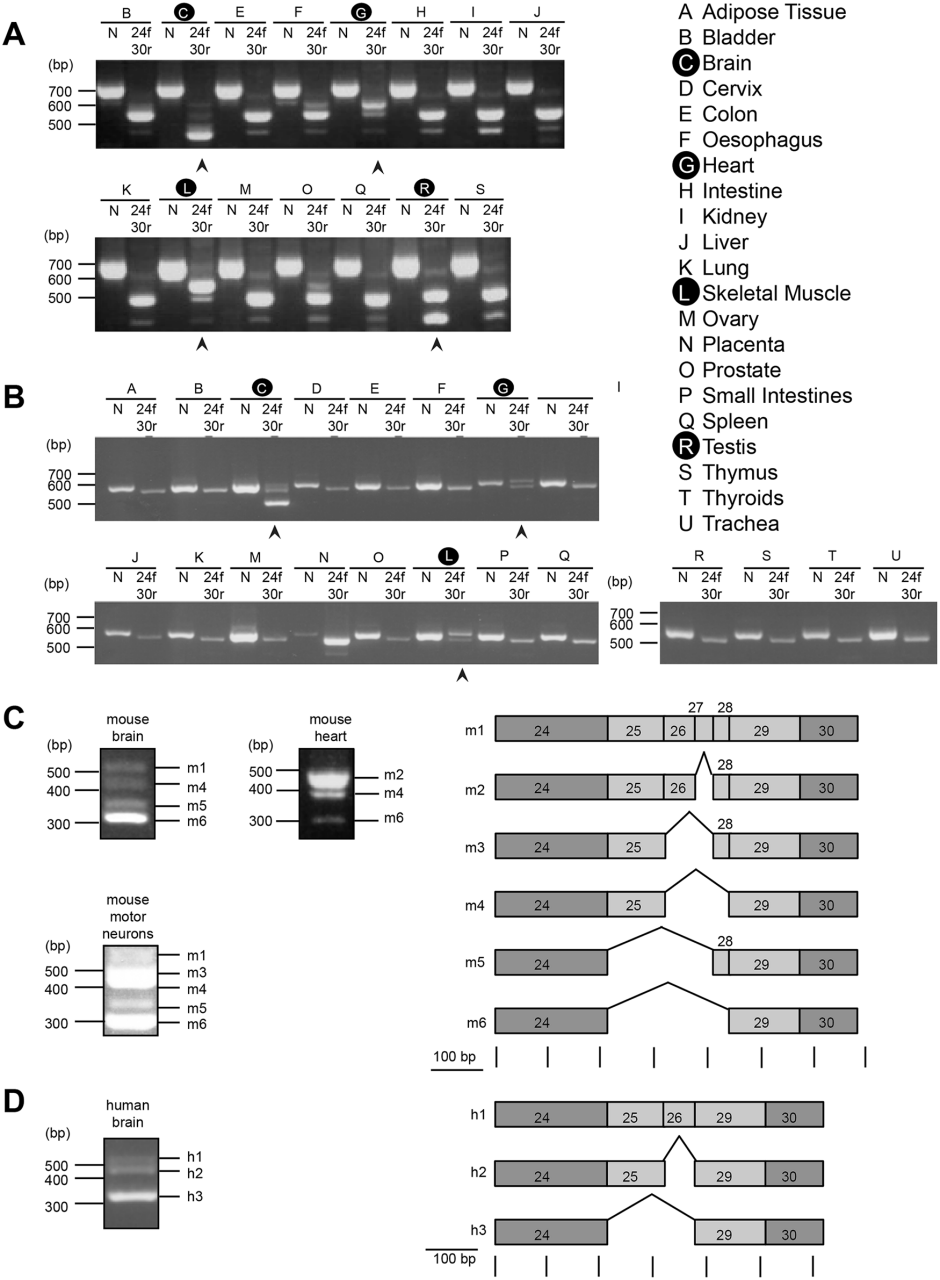
Expression of alternative splice isoforms of Kidins220 in adult mouse and human tissues. (A-B) RT-PCR analyses for exons encoding the amino-terminus of Kidins220 and between exons 24 and 30 (24f-30r) were carried out on adult mouse (A) and human (B) tissue panels. Tissues are labelled by capital letters. N indicates PCR products obtained using primers designed to recognise exons 3 and 8 in mouse, and exon 9 and 13 in human. 24f-30r indicates samples obtained by amplification with primers recognising exons 24 and 30. Arrowheads point to samples in which a specific alternative splicing pattern was detected. (C-D) Schematics of Kidins220 splice isoforms identified in mouse heart and brain (C) and human brain (D) in the region encoded by exons 24 to 30.

To verify the expression of alternative splice isoforms of Kidins220 between exon 24 and 29, we sequenced Kidins220 in mouse (Fig 1A) and human (Fig 1B) RNA tissue panels. We extracted RNA from wild type adult mouse tissues and compared them to a commercial RNA panel from a pool of healthy adult human tissues. The RNA from both panels was reverse-transcribed, amplified using specific Kidins220 primers (see Materials and Methods) and analysed by standard gel electrophoresis (Fig 1A and 1B). As a control (N) of the quality of our cDNA and Kidins220 expression, we designed primers specific for invariant regions of Kidins220, such as exons 3 and 8 in the case of mouse samples (Fig 1A) and for exons 9 and 13 for human samples (Fig 1B). To investigate potential alternative splicing patterns, we selected primers specific for exons 24 and 30 for both panels. As shown in Fig 1, we observed a mouse-specific and a human-specific “default pattern”, which is identical in several tissues. In the mouse tissue panel, we obtained three different bands ranging between 400 and 700 bp (samples B, E-F, H-K, M-Q and S; Fig 1A), whereas the human “default pattern” comprises only a single band migrating between 500 and 600 bp (samples A, B, D-F, I-O and P-U; Fig 1B). However, this pattern differed in brain, heart and skeletal muscle (indicated by arrowheads), both in mouse and human tissues. Additionally, a different splice pattern was detected in mouse testis (Fig 1A, arrowhead).

Since systemic Kidins220 knockout caused severe phenotypes in the nervous and cardiovascular systems leading to embryonic death [24, 25], we determined the sequences of these putative splice isoforms in mouse brain and heart (Fig 1C), and in the human brain (Fig 1D). To this end, we extracted the cDNA from the specific bands and sequenced the putative splice isoforms. We found six alternative Kidins220 variants in mouse tissues, which we named isoforms m1-m6 and which are shown as a schematic in Fig 1C. In human brain, we identified three different alternative splice isoforms of Kidins220 (h1-h3), which are shown in Fig 1D. Sequence comparison of mouse, human and rat splice isoforms between exon 24 and 30 is shown in S3 Fig.

### Kidins220 undergoes alternative terminal exon splicing in mouse and human tissues

**Fig 2:**
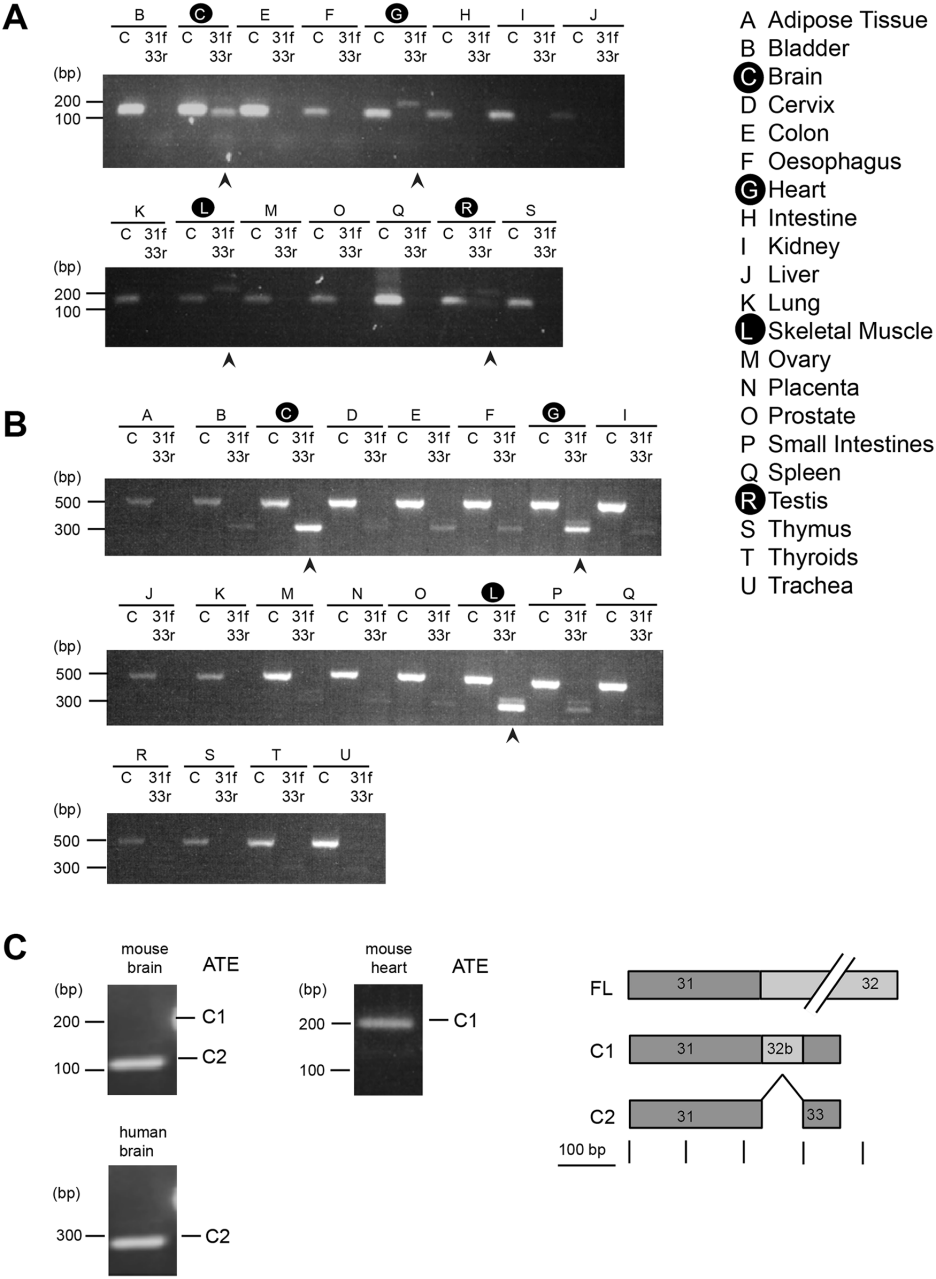
Kidins220 alternative terminal exons in mouse and human tissue. (A-B) RT-PCR analysis for the carboxy-terminal endings of Kidins220 (exon 32 and exon 33) was carried out on an adult mouse (A) and human (B) tissue panels. Capital letters indicate the different tissues. 31f and 33 rev indicate amplification with primers against exons 31 and 33. Arrowheads point out samples where alternative terminal exon (ATE) splicing for Kidins220 is detected. (C) PCR products obtained using primers designed to recognise exons encoding the carboxy-terminus of Kidins220 (exons 31/32 and 32 in mouse and exon 31 and 32 in human).

To investigate potential ATE splicing, we analysed cDNA from mouse and human tissues using specific primers. According to our bioinformatics analysis (S1 and S2 Fig), exon 32 could be potentially spliced out and replaced by a much shorter exon 33. To test this hypothesis, cDNA was amplified using primers designed to recognise mouse (Fig 2A) and human (Fig 2B) exons 31, 32 and 33. In both tissue panels, primers recognising exon 32 were used as a positive control (indicated as ‘C’), whereas bands appearing in samples incubated with primers 31f and 33rev indicate whether ATE splicing has occurred in certain tissues.

Strikingly, we found evidence of ATE only in tissues not presenting “default patterns” and therefore where specific splice variants in the region between exons 24 and 29 were detected. Accordingly, we observed ATE in mouse and human brain, heart, skeletal muscle (Fig 2A and 2B) and mouse testis (Fig 2A). Sequencing of the different amplified bands in Fig 2 revealed two different alternative endings: ATE C1, which encodes a small portion of exon 32 (the first 81 bp of exon 32) and exon 33, and ATE C2 which encodes exon 33 only (Fig 2C).

### Alternative splice variants of Kidins220 are translated into specific proteins in embryonic and adult mouse brain

The pattern of RNA expression (Figs 1 and 2) and the computational analysis (S1–S3 Figs) suggest that Kidins220 splice variants displays a complex tissue-specific distribution yielding the translation of several proteins of different length and domain composition. To test this hypothesis, we generated rabbit polyclonal antibodies directed against alternative splice isoforms m1, m6 and ATE C2 (see Materials and Methods). These antibodies were tested for their ability to specifically recognise different Kidins220 splice variants (S4 Fig). To facilitate this, antibodies m1 and m6 were pre-incubated with vehicle or with an excess of m1 and m6 peptides prior to western blotting using extract from embryonic (E) or adult (A) mouse brain (S4 Fig). The antibody m1 recognises a single band at 220 kDa in both adult and embryonic extracts, whereas the antibody m6 revealed a band at the same molecular weight only in the embryonic sample.

To test the specificity of the ATE C2 antibody, we opted to use GST-fusion proteins with exon 32 (GST-mEx32; canonical Kidins220) or exon 33 (GST-mEx33; correspondent to ATE C2). As shown in S4 Fig., an antibody directed against ATE C2 detects GST-mEx33, but not GST-mEx32, which is instead recognised by an antibody (GSC16), raised against the carboxy-terminus of full-length Kidins220 [34]. Importantly, the signals detected by the m1, m6 and ATE C2 antibody are specific, since they are blocked by pre-incubation with the immunising peptides (S4 Fig).

**Fig 3:**
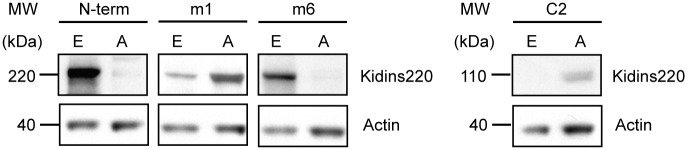
Kidins220 alternative splice isoforms are translated into protein isoforms in embryonic and adult mouse brain. Western blots of embryonic day 18.5 (E) and adult (A) brain lysates show evidence of protein translation of Kidins220 variants containing: amino-terminus (N-term), Kidins220 isoform 1 (m1), Kidins220 isoform 6 (m6) and exon 33 (C2). Actin was used a loading control.

We then used these specific antibodies to test for protein expression using embryonic and adult mouse brain lysates (Fig 3). Splice isoform m1, which contains exon 24 to 30 and corresponds to full-length Kidins220, was mainly expressed in adult brain, as hardly any expression was detected in embryonic tissue. Isoform m6, however, shows an opposite distribution pattern with high expression levels during embryogenesis and an absence in adult brain (Fig 3). In contrast, the splice variant ATE C2 was found only in adult brain tissue (Fig 3), mimicking the expression pattern seen for splice isoform m1. As an internal control, we used an antibody raised against the amino-terminus of Kidins220 (KNA), which confirmed the higher expression levels of Kidins220 in the nervous system during embryonic development compared to adulthood (N-term; Fig 3) [28, 34].

### Alternative splicing of Kidins220 is developmentally regulated

**Fig 4:**
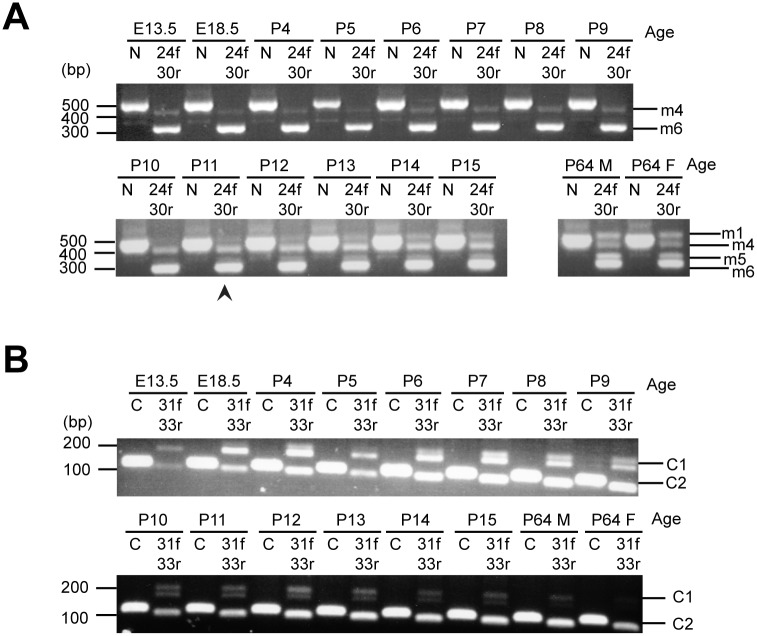
Developmental-specific expression of Kidins220 alternative splice isoforms in mouse brain. (A-B) RT-PCR analyses for exons encoding the amino-terminus of Kidins220 and between exons 24 and 30 (A), and for alternative terminal exon splicing (B) were carried out on mouse brains at different developmental stages, starting from embryonic stage E13.5 up to postnatal stage P64. The analysis at the latter stage includes adult male (M) and female (F) brains. N indicates PCR products obtained using primers designed to recognise exons 3 and 8. 24f-30r indicates samples obtained by amplification with primers recognising exons 24 and 30 (A). C indicates PCR products obtained using primers designed to recognise exons 31/32 and 32. 31f-33r indicates samples obtained by amplification with primers recognising exons 31 and 33 (B). The arrowhead points to appearance of Kidins220 isoform m1 (full-length) at postnatal stage P11 (A).

Since the expression levels of Kidins220 splice variants undergo dramatic changes in embryonic and adult mouse brain, we sought to investigate their regulation during development. We therefore extracted RNA from mouse brains starting at E13.5 until postnatal day 64 (P64), and after reverse transcription, amplified these samples with primers designed to recognise exon 24 and 30 (Fig 4A) and exon 31 and 33 (Fig 4B).

As shown in Fig 4A, E13.5 mouse brains express isoforms m4 and m6 until P10. However, from P11 onwards isoforms m1, m4, m5 and m6 become dominant and their expression sustained until adulthood. Remarkably, gender did not have any impact on the alternative splicing of Kidins220 in the adult mouse brain (M, male; F, female; Fig 4A).

In contrast, ATE C1 and C2 showed very faint bands at E13.5 and both increased until P9. From P10 onwards ATE C1 decreased slowly and was not found in adult brain tissue anymore, whereas C2 displays stable expression levels (Fig 4B).

### Different neuronal populations display specific Kidins220 splice variants

The alternative splice variants of Kidins220 in the nervous system and their developmental regulation begs the question whether distinct neuronal populations differ in terms of their repertoire of Kidins220 isoforms and whether the presence of these variants changes during neuronal differentiation.

**Fig 5:**
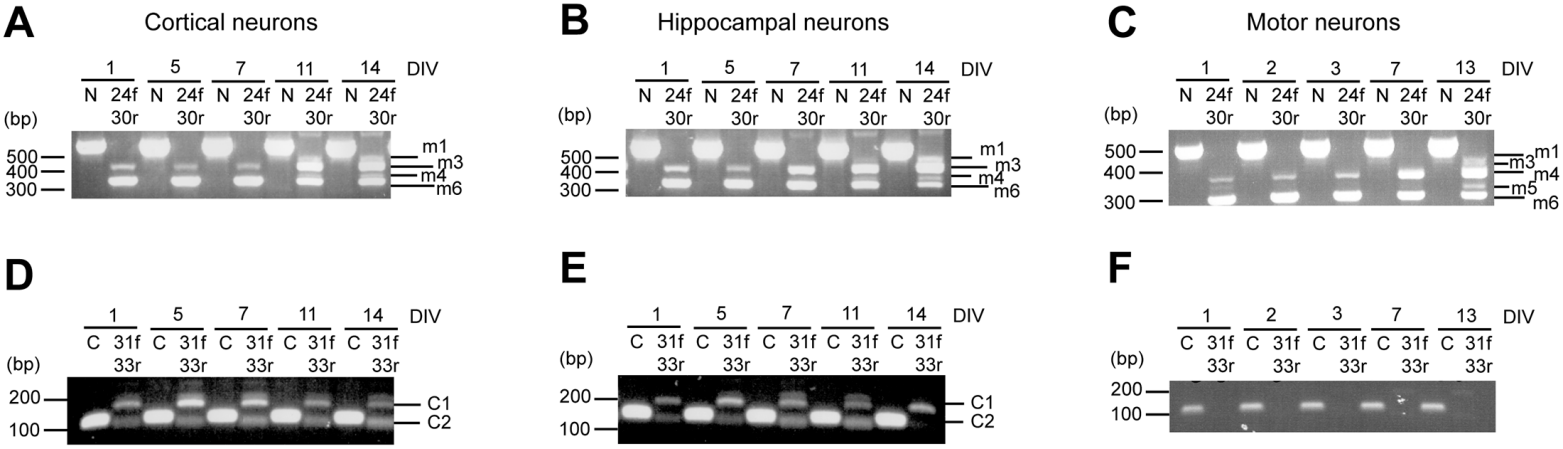
Different neuronal populations express specific Kidins220 splice isoforms. (A-F) RNA extracted from cortical (A, D), hippocampal (B, E) and motor neuron (C, F) primary cultures was reverse transcribed into cDNA. N indicates PCR products obtained using primers designed to recognise exons 3 and 8 of Kidins220. 24f-30r indicates samples obtained by amplification with primers recognising exons 24 and 30 (A-C). C indicates PCR products obtained using primers designed to recognise exons 31/32 and 32. 31f-33r indicates samples obtained by amplification with primers recognising exons 31 and 33 (D-F). Note the absence of ATE splicing isoforms C1 and C2 in primary motor neuron cultures.

To address this point, we prepared primary cortical and hippocampal cultures (from E18.5 embryos) and primary motor neurons (from E13.5 embryos), and extracted RNA from these cells at different days *in vitro* (DIV). Strikingly, we found isoforms m4 and m6 from DIV 1 to DIV 7 in all neuronal populations (Fig 5A–5C). Cortical and hippocampal neurons show similar Kidins220 expression patterns between exons 24 and 30: from DIV 11 onwards, both populations express isoforms m1 and m3 in addition to isoforms m4 and m6. Motor neurons, however, show the highest variety of Kidins220 splice isoforms in the nervous system, with the expression at DIV 13 of isoform m5 in addition to those previously described for cortical and hippocampal neurons (Fig 5C).

Also the occurrence of ATE variants differs between the three neuronal populations (Fig 5D–5F). In cortical neurons, the ATE C2 isoform appears to gradually increase during *in vitro* maturation, whereas the ATE C1 has its strongest expression between DIV 5 and 7 and then decreases to basal levels at DIV 14 (Fig 5D). Hippocampal neurons show a different pattern: the ATE C2 isoform displays a gradual increase until DIV11, but is absent at DIV 14. In contrast, ATE C1 is constantly present until DIV 14 (Fig 5E). Strikingly, motor neurons appear to lack any significant ATE splicing (Fig 5F). These differences in the composition and timing of expression of Kidins220 may reflect the distinct requirements of these neuronal populations in terms of neurotrophin signalling for their differentiation and/or survival.

### BDNF and NGF modulate Kidins220 alternative splicing

Kidins220 binds to Trk receptors and p75^NTR^, and is a downstream target of Trk activation [37]. In light of these findings and the results shown in Fig 5, we sought to investigate whether stimulation of neurotrophin receptors by BDNF and NGF would have an impact on Kidins220 alternative splicing.

**Fig 6:**
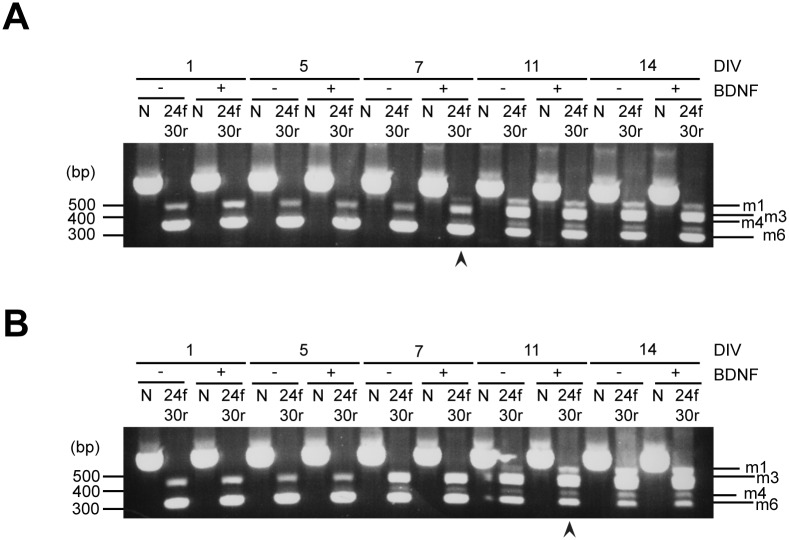
BDNF accelerates the appearance of full-length Kidins220 in cortical and hippocampal primary neurons. (A-B) Primary cortical (A) and hippocampal (B) neurons were prepared from E18.5 mouse embryos and half of each culture was plated with medium containing 100 ng/ml BDNF. RNA was extracted at different time points and reverse transcribed. N indicates PCR products obtained using primers designed to recognise exons 3 and 8. 24f-30r indicates samples obtained by amplification with primers recognising exons 24 and 30. (A) Arrowheads point to early appearance of Kidins220 isoform m1 (full-length) in BDNF treated cultures compared to non-treated cultures.

We plated primary cortical and hippocampal cultures with or without BDNF (100 ng/ml) and collected their RNA at different time points (Fig 6). In parallel, we plated PC12 cells and stimulated them with NGF (100 ng/ml) for 72 h (S5 Fig). Samples were reverse transcribed and amplified with primers recognising exons 24 and 30 and exons 31 and 33. BDNF accelerated the appearance of the m1 isoform in both primary cortical and hippocampal cultures compared to non-stimulated samples. In cortical neurons, isoform m1 appeared at DIV 7 in stimulated samples (Fig 6A, arrowhead), whilst only at DIV 11 in absence of BDNF. In hippocampal cultures, BDNF induced the robust expression of isoform 1 at DIV 11 (Fig 6B, arrowhead), whereas a band corresponding to this Kidins220 variant only appeared in significant amounts at DIV 14 in non-stimulated samples.

Upon stimulation with NGF, PC12 cells undergo a process of neuronal differentiation, which results in neurite outgrowth and expression of several synaptic markers [38]. Treatment with NGF led to the appearance in PC12 cells of two additional Kidins220 splice isoforms in the central region (exons 24 and 29) after 48 h of differentiation (S5 Fig). However, neither BDNF nor NGF had any effect on ATE splicing in these cells in any of the tested conditions (data not shown).

### Kidins220 splice isoforms display different cellular localisations

**Fig 7:**
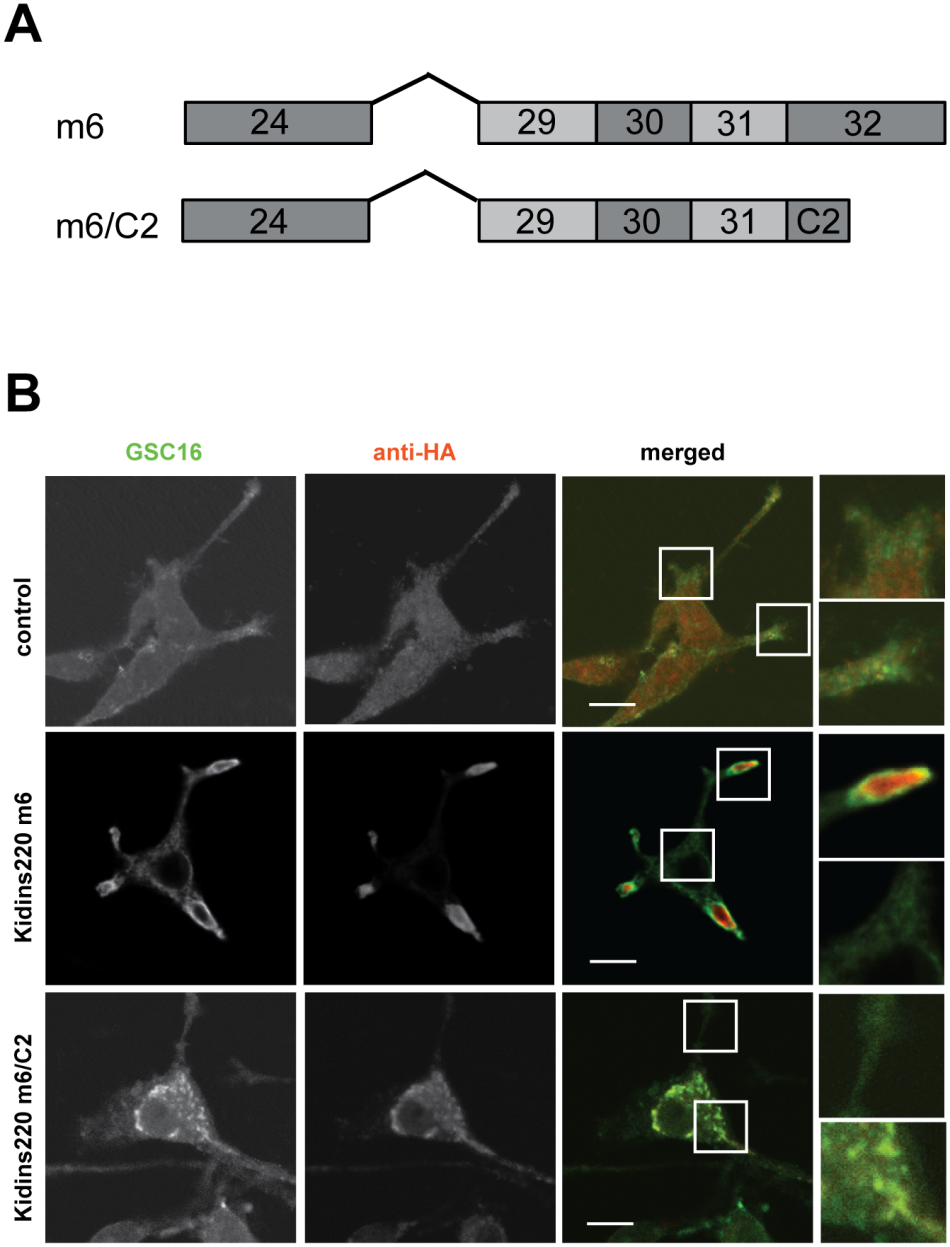
Distinct Kidins220 splice isoforms display specific cellular localisations. (A) Schematics of Kidins220 splice isoform m6 and Kidins220 ATE m6/C2 (from Exon 24 onwards) used for transfection of PC12 cells in Fig 7B. (B) PC12 cells were transfected with HA-tagged Kidins220 isoform m6, isoform m6/C2 or with Tet-ON pLVX vector only (control) and after 6 h stimulated with doxycycline and differentiated for 48 h with NGF. Full-length Kidins220 was detected using a polyclonal antibody directed against the carboxy-terminus of Kidins220 (GSC16 antibody; in green). An anti-HA antibody was used to stain Kidins220 isoforms m6 and m6/C2 (in red). The gain of the red channel was enhanced equally for cells overexpressing isoform m6/C2 and control cells, whilst it was tuned down for PC12 cells transfected with isoform m6 to adjust for the higher expression levels of this Kidins220 variant. Boxed areas of the merged images are magnified on the right. Representative pictures were chosen from three different experiments. Scale bars, 10 μm.

NGF treatment is known to change the cellular distribution of Kidins220 from the plasma membrane to the neurite tips in differentiated PC12 cells [34], a process which relies on the recruitment of kinesin-1 to the carboxy-terminal domain of Kidins220 and is required for physiological TrkA signalling [31]. To determine potential differences in the localisation of Kidins220 isoforms in PC12 cells, we selected isoform m6 and m6/C2 (Fig 7A), which are endogenously expressed in these cells (data not shown). We transfected HA-tagged constructs of isoforms m6, isoform m6/C2 and pLVX TetON vector only (control) into undifferentiated PC12 cells, which were then treated with NGF for 48 h (Fig 7) and into HEK cells (S6 Fig). Cells were fixed and stained for exon 32 (including endogenous and exogenous Kidins220 isoforms m6) using an antibody directed against the carboxy-terminus of Kidins220 (GSC16) or total Kidins220 level (KNA) and for the exogenous variants using an anti-HA antibody (Fig 7B and S6 Fig, respectively).

As expected, staining of control PC12 cells with the GSC16 antibody revealed an accumulation of Kidins220 at the tips of growing neurites (Fig 7B) and KNA staining of total Kidins220 was found in the plasma membrane of HEK cells (S6 Fig). Transfected Kidins220 isoform m6 displays a similar distribution, with areas of extensive colocalisation between exogenous and endogenous Kidins220 at the level of neurite extensions in PC12 cells (Fig 7B) [31, 39]. In HEK cells, we also found colocalisation between endogenous and exogenous Kidins220 isoform m6 (S6 Fig). In contrast, isoform m6/C2 was found to distribute in intracellular puncta in the cell body in both PC12 and HEK cells, with negligible accumulation in the neurite tips in PC12 cells. Expression of these Kidins220 variants in primary wild type hippocampal neurons yields similar results, with isoform m6 distributed in axon and dendrites, whereas isoform m6/C2 is retained in the soma (S7 Fig).

Importantly, overexpression of this ATE splice variant alters the distribution of endogenous full-length Kidins220, which could no longer be detected in the neurite tips or at the plasma membrane in both both PC12 and HEK cells (Fig 7B and S6 Fig).

### Kidins220 ATE C2 leads to increased TrkA expression

Because Kidins220 forms a ternary complex with the TrkA receptor and p75^NTR^ [27], we then investigated whether the overexpression of Kidins220 isoform m6 and m6/C2 (Fig 7A) alters the cellular distribution and/or expression levels of TrkA and p75^NTR^, which are both endogenously expressed in PC12 cells.

**Fig 8:**
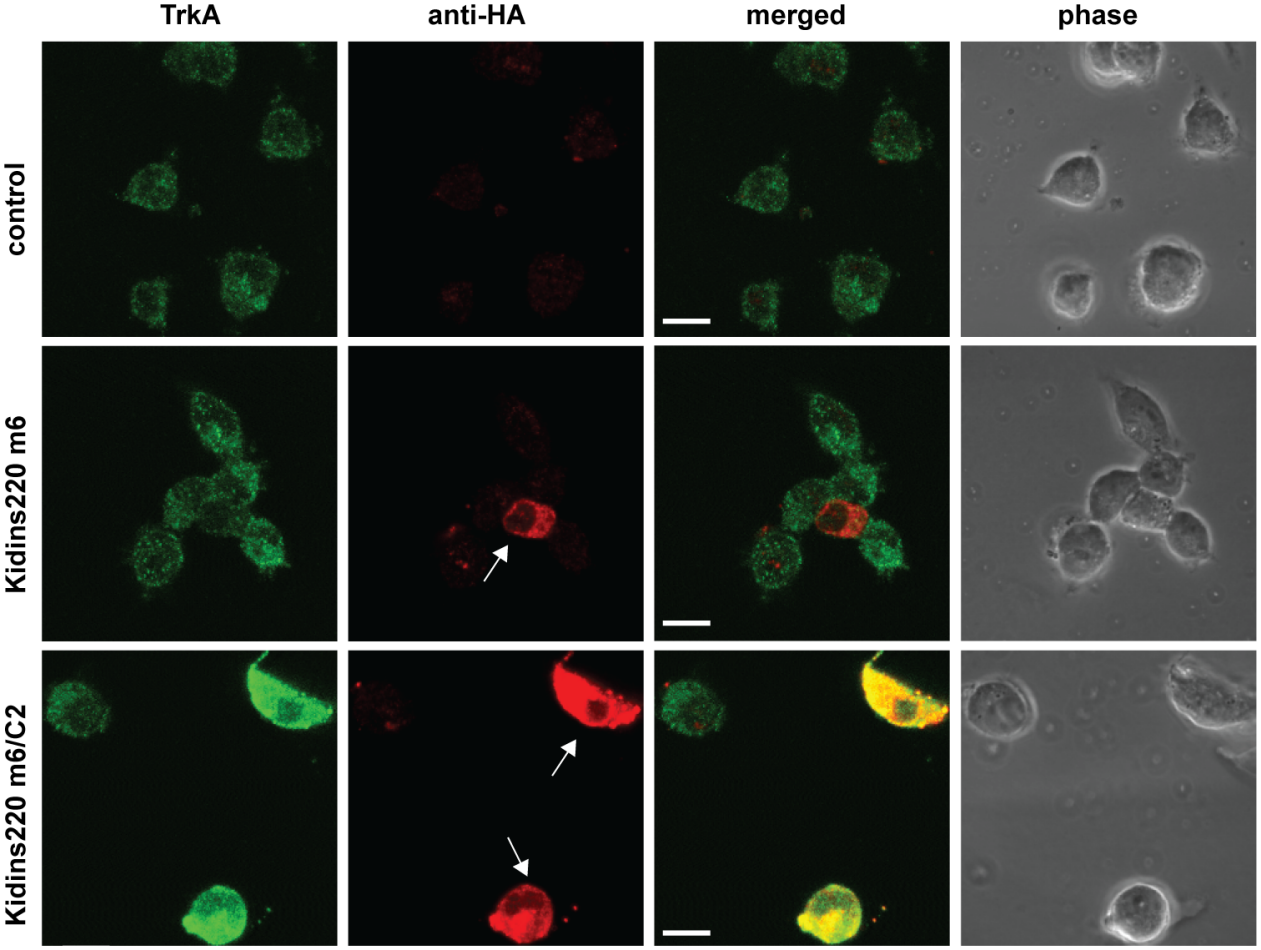
Kidins220 isoform m6/C2 leads to increased TrkA expression. PC12 cells were transfected with HA-tagged Kidins220 isoform m6, isoform m6/C2 or with Tet-ON pLVX vector only (control) and after 6 h stimulated with doxycycline for 48 h. Samples were stained for TrkA (in green) and for HA (in red). Arrowheads indicate transfected cells. All channels were adjusted equally to accommodate the increase in TrkA levels upon m6/C2 expression. Representative pictures were chosen from three different experiments. Scale bars, 10 μm.

As shown in Fig 8, overexpression of the full-length Kidins220 isoform m6 did not alter TrkA expression levels, which were comparable to those found in untransfected cells. However, overexpression of the short isoform m6/C2 lead to a drastic increase of TrkA expression levels (Fig 8), suggesting a specific role for the long carboxy-terminus of Kidins220 in TrkA homeostasis. Similar results have been obtained using HEK cells stably transfected with TrkA (S8 Fig). Importantly, this regulation appeared to be specific, since overexpression of either isoforms did not affect p75^NTR^ levels (data not shown).

## Discussion

In this work, we describe the characterisation of several alternative splice isoforms of Kidins220, a scaffold protein for NT receptors and VEGFRs. Two major splicing regions have been identified by our work: the first maps to the central portion of Kidins220 encoded by exons 24 to 29, whilst the second results from the alternative terminal exon (ATE) splicing of exon 32 and a previously uncharacterised exon 33. Kidins220 splicing is regulated in space and time, and is highly conserved in mouse and humans. Importantly, these splicing events determine the acquisition (or the loss) of important protein-protein interaction domains, which might cause additional and still poorly understood structural changes. This novel mechanism has therefore the potential to regulate Kidins220 functions in specific tissues during development and adulthood.

We confirmed bioinformatics evidence on the existence of Kidins220 splice variants by showing their presence in different mouse, rat and human cells and tissues at both RNA and protein levels. By investigating the occurrence of Kidins220 RNA in mouse and human tissue panels, we found in addition to a conserved “default pattern” of Kidins220 isoforms, which remains constant in a variety of tissues, alternatively spliced isoforms in brain, heart, skeletal muscle and in mouse testis (Figs 1 and 2). Similar splice isoforms were found also in rat PC12 cells (S3 Fig). We were able to detect six different splice isoforms in the central region of Kidins220 in mice (m1-6; Fig 1C) and rat (r1-4, 6,7; S3 Fig), but only three in humans (h1-3; Fig 1D). This is due to the fact that exons 27 and 28 are undetectable in the human genome (data not shown), thus limiting the splicing diversity in humans. We also found that mouse testis had a different splicing pattern to other tissues, whereas human testis did not show any detectable difference. To date, nothing is known about the role of Kidins220 in testis and how this alternative splice isoform pattern could affect the functionality of this organ in mice. In Kidins220^lox/lox^ mice, a cDNA-polyA cassette encoding full-length Kidins220 corresponding to isoform m1, was inserted downstream of exon 16 (25). As a result, these animals only express this Kidins220 splice variant. Although Kidins220^lox/lox^ females are viable and lack overt behavioural phenotypes (25), Kidins220^lox/lox^ males are sterile (data not shown), suggesting that the expression of the Kidins220 m1 isoform is not sufficient for physiological testis function, which requires the correct pool of Kidins220 splice variants (Fig 1A).

Kidins220 plays an important role in the development of the nervous and the cardiovascular systems [24, 25], where it binds to NT and VEGF receptors. It is therefore not surprising to find several Kidins220 variants in heart and brain. While Kidins220 splice isoforms between exon 24 and 29 change during brain development, they remain invariant in heart, with a characteristic isoform pattern (data not shown). We believe that this specific pool of Kidins220 isoforms is required for a fully functional cardiovascular system, since at a more careful phenotypic examination, Kidins220^lox/lox^ mice, which only express the Kidins220 m1 spice variant under the endogenous promoter [24, 25], display heart abnormalities, which include atrial dilation (data not shown).

Whilst potential binding partners for the region encoded by exons 25 to 29 remain unknown, Kidins220 isoform m6/C2 lacks its kinesin binding motif (KIM), its p75^NTR^ binding site and its PDZ binding domain. Kidins220 is responsible for neurite outgrowth upon BDNF stimulation [25] and is found in the neurite tips of differentiating PC12 cells [34]. To reach neurite tips, Kidins220 relies on its KIM domain to bind kinesin 1 via the kinesin light chain (KLC1/2). This microtubule-dependent motor mediates the transport of Kidins220 to the microtubule plus end, which is located distally in differentiated axons and dendrites. Crucially, neurite tips are active sites for NT signalling [31]. Consequently, this process might be important during the development of a functional neuronal network, but less so once neurons are fully differentiated. This prediction was confirmed by our experimental evidence showing that ATE splicing occurs mainly during adulthood and after several days *in vitro* in cortical and hippocampal cultures. Interestingly, Kidins220 RNA did not display any ATE splicing in motor neurons over 13 days *in vitro* (Fig 5F), suggesting that the carboxy-terminus of Kidins220 encoded by exon 32 is required for the development and/or maintenance of this type of neuron, e.g. to ensure physiological neurotrophin signalling by modulating the distribution and levels of Trk and p75^NTR^ receptors.

Hardly anything is known about the role of Kidins220 in adult tissues, since ablation of Kidins220 is embryonic lethal [25]. The study of Kidins220 splice variants might represent a novel strategy to unravel these still unknown functions. For example, alterations in the region between exon 24 and 29 might lead to structural changes, or alter the recruitment of yet undiscovered binding partners. Similarly, whereas ATE splicing seems to drive an overall loss of function by the loss of various binding sites encoded by exon 32, nothing is known about possible gain of functions, which may be mediated by exon 33-specific binding partners. Crucially, it remains to be elucidated which combinations of ATE and central domain alternative splicing are expressed in different tissues, how they might be changing over time and which specific role they might have.

In differentiated PC12 cells and hippocampal neurons, we showed different localisations of full-length Kidins220 isoform m6 with or without its ATE splicing C2 (Fig 7 and S7 Fig). Whilst Kidins220 with a long carboxy-terminus encoded by exon 32 (aminoacid 1–1793) co-localises with endogenous Kidins220 (Fig 7B), overexpression of its shorter version in which this exon has been replaced by exon 33 (Fig 7A, m6/C2; aminoacid 1–1420), seems to act in a dominant-negative fashion by altering the distribution of endogenous Kidins220. As shown in Fig 7B and S7 Fig, m6/C2 displays a punctate localisation in the cell body, and appears to sequester endogenous Kidins220 in these puncta (Fig 7B). This alternative distribution is likely to be driven by the loss of the KIM domain, and the inability of m6/C2 to recruit kinesin-1, and possibly other motor proteins [31], thus impairing its transport to the neurite tips. However, this change in localisation may be due to novel binding partners of the m6/C2 isoform, which may restrict Kidins220 distribution to the cell body. In this regard, given the role of Kidins220 in NT signalling, its splice variants might play different roles in signalling endosomes [40] or recycling endosomes [41], whose in depth analysis will be provided in future studies.

Since the m6/C2 splice isoform lacks its p75^NTR^-binding site, it is neither expected to be transported together with p75^NTR^, nor to form a ternary complex with TrkA and p75^NTR^. We found that this isoform increased the cellular level of TrkA (Fig 8 and S8 Fig), but not of p75^NTR^. The molecular mechanism responsible for this strong increase of TrkA expression remains yet to be determined. Kidins220 isoform m6/C2 might sequester TrkA receptors in the cell body and prevent its targeting to lysosomes or other degradation pathways [42, 43]. This may be due to the interaction of the retromer/SNX27 complex to full-length Kidins220 via its PDZ binding domain [32], which is lost in Kidins220 m6/C2 splice variants. Alternatively, Kidins220 m6/C2 might promote TrkA recycling or regulate its translation. It is therefore likely that Kidins220 regulates NT signalling and function not only by modulating the intracellular trafficking of NT receptors but also by directly affecting receptor homeostasis.

Previous studies have shown that increasing Kidins220 levels lead to the decreased association of p75^NTR^ with TrkA [27]. In this light, Kidins220 ATE splicing could provide an effective tool to fine tune NT signalling throughout development. On the other hand, NTs may influence Kidins220 function by controlling the kinetics of expression of its isoforms, as shown by the accelerated appearance of isoform m1 upon BDNF stimulation in cortical and hippocampal neurons (Fig 6), or the expression of additional Kidins220 splice variants in PC12 cells upon NGF stimulation (S5 Fig). This feedback mechanism may represent an important pathway controlling how a target cell responds to NTs by adapting its repertoire of Kidins220 isoforms, and as a consequence, the strength of NT signalling output. NT signalling would therefore not only depend on the regulation of the alternative splicing of the NT ligand (e.g. BDNF) [44], and its receptors (e.g. TrkB) [18], but also on the compendium of Kidins220 isoforms generated by alternative splicing.

In summary, Kidins220 is a crucial protein for neuronal and cardiovascular development, which by undergoing alternative splicing, might adapt to specific requirements during development, such as the varying intracellular distribution of growth factor receptors or the modulation of specific signalling events. Future studies will determine the exact role of Kidins220 isoforms in this process, and its regulation in the maintenance of the nervous and cardiovascular systems during adulthood.

## Supporting Information

S1 Table Kidins220 splice isoforms and their accession numbers. (PDF)

S2 Table Kidins220 alternative terminal exons (ATE) and their sequences. (PDF)

S1 Fig Genomic view of mouse Kidins220 intron-exon structure. Schematic showing mouse Kidins220 exon-intron structure between exons 23 and 33 (NM_001081378) (www.genome.ucsc.edu). Horizontal lines represent different mouse ESTs. Numbered red boxes mark Kidins220 exons.
(PDF)

S2 Fig Genomic view of human Kidins220 intron-exon structure. Schematic showing human Kidins220 exon-intron structure between exons 25 and 33 (www.genome.ucsc.edu). Horizontal lines represent different mouse ESTs. Numbered red boxes mark Kidins220 exons.
(PDF)

S3 Fig Genomic view and sequence comparison of mouse, rat and human Kidins220 splice variants. Schematic showing: (A) mouse Kidins220 (NM_001081378) exon-intron structure on chromosome 12 (Chr12), (B) area of interest between exons 24 and 30 and (C) sequence alignment of the mouse (m1-6), human (h1-3) and rat (r1-4, 6, 7) splice variants identified in this study and during the cloning of rat Kidins220 [34]. Vertical green rectangles and numbering refer to mouse exons.
(PDF)

S4 Fig Specificity of antibodies recognising Kidins220 splice isoforms m1, m6 and C2. (A) Antibodies targeting the alternative splice isoforms m1 and m6 of mouse Kidins220 were tested by peptide competition in embryonic day 18.5 (E) and adult (A) brain lysates. Western blots were probed with the indicated antibodies with or without pre-incubation with the specific peptides (100 μM). (B) Recombinant GST fusion proteins of exon 32 and exon 33, together with control GST, were resolved in SDS-PAGE and either stained with Coomassie Blue R (left panel) or transferred on nitrocellulose and probed with the indicated antibodies (right panels). The rabbit polyclonal antibody GSC16 [31] recognised GST-exon 32, but not GST-exon 33, whereas the rabbit polyclonal antibody C2 specifically stained GST-exon 33. This signal was abolished by pre-incubating this antibody with an excess of the immunising peptide (100 μM; C2+peptide).
(PDF)

S5 Fig Induction of Kidins220 alternative splice isoforms by NGF in PC12 cells. PC12 cells were maintained in control medium or treated with 100 ng/ml NGF for the indicated times. RNA was then extracted and reverse transcribed. N indicates PCR products obtained using primers designed to recognise exons 3 and 8. 24f-30r indicates samples obtained by amplification with primers recognising exons 24 and 30.
(PDF)

S6 Fig Isoforms m6 and m6/C2 show different localisation in HEK cells. HEK cells were transfected with Tet-ON pLVX vector only (control), HA-tagged Kidins220 isoform m6 or isoform m6/C2 and stimulated with doxycycline for 24 h. The localisation of total Kidins220 was revealed using a polyclonal antibody directed against the amino-terminus (KNA antibody; in green). An anti-HA antibody was used to stain Kidins220 isoforms m6 and m6/C2 (in red). Arrows indicate distinct Kidins220 isoform patterns for isoforms m6 (plasma membrane and outgrowths) and m6/C2 (puncta in the cell body). Scale bars are 10 μm for the control and the Kidins220 m6/C2 panels, and 20 μm for the Kidins220 m6 panels.
(PDF)

S7 Fig Different Kidins220 splice isoforms display specific cellular localisations. Hippocampal neurons were transfected with a Tet-ON pLVX vector encoding HA-tagged Kidins220 isoform m6 or isoform m6/C2 and after 4 h stimulated with doxycycline. An anti-HA antibody was used to stain Kidins220 isoforms m6 and m6/C2 (in red) after 48 h. The neuronal cytoskeleton was stained with TUJ1 (in green). Arrows indicate the presence of Kidins220 isoforms m6 in neurites. The arrowhead points to the somatic accumulation of the m6/C2 splice variant. Scale bars, 10 μm.
(PDF)

S8 Fig Kidins220 isoform m6/C2 leads to increased TrkA expression in HEK cells. HEK-TrkA cells were transfected with a Tet-ON pLVX vector encoding HA-tagged Kidins220 isoform m6 or isoform m6/C2. Samples were stained for TrkA (in green) and for HA Kidins220 (in red). Arrowheads indicate transfected cells. All channels were adjusted equally to accommodate the increase in TrkA levels upon m6/C2 expression. Scale bars, 20 μm.
(PDF)
