# Supplementary material for: Novel Kidins220/ARMS Splice Isoforms: Potential Specific Regulators of Neuronal and Cardiovascular Development
Source: PLoS One. 2015 Jun 17;10(6):e0129944. doi: 10.1371/journal.pone.0129944 (PMC4470590; doi:10.1371/journal.pone.0129944)
Supplement: S1 Table — (PDF) [file pone.0129944.s001.pdf]

S1 Table. Kidins220 splice isoforms and their accession numbers.

Mouse Kidins220 Splice Isoform	Genbank Accession Number
m1	KJ812113
m2	KJ812114
m3	KJ812115
m4	KJ812116
m5	KJ812117
m6	KJ812118

Human Kidins220 Splice Isoform	Genbank Accession Number
h1	KJ812119
h2	KJ812120
h3	KJ812121

Rat Kidins220 Splice Isoform	Genbank Accession Number
r1	KR081254
r2	KR081255
r3	KR081256
r4	KR081257
r6	KR081258
r7	KR081259
