# Supplementary material for: Novel Kidins220/ARMS Splice Isoforms: Potential Specific Regulators of Neuronal and Cardiovascular Development
Source: PLoS One. 2015 Jun 17;10(6):e0129944. doi: 10.1371/journal.pone.0129944 (PMC4470590; doi:10.1371/journal.pone.0129944)
Supplement: S2 Table — (PDF) [file pone.0129944.s002.pdf]

S2 Table. Kidins220 alternative terminal exons (ATE) and their sequences.

Mouse Alternative Terminal Exons	Sequences
C1	TCACAAACTCGCAGAACCCCAAGTCTCTCGAGTCTCAATTCCCAGGACTCCAGTATTGAAATTTCAAAGCTTACTGATAAGGAAGAAAATGCCGCCCCTGTGCTAGATAGACAGCGGTTCAGAAGATCCAGTTTAAACTGAG
C2	GAAGAAAATGCCGCCCCTGTGCTAGATAGACAGCGGTTCAGAAGATCCAGTTTAAACTGAG

Human Alternative Terminal Exon	Sequence
C2	GAAAATGCTGGCCTTGAGATAGATAGCTCTTCAGAGGATCCAGATTAAACTAAGTTCGAGTCACTTCTCACAGTTGACTTCCTCACCAAATAAT
