# Supplementary figures and images for: Novel Kidins220/ARMS Splice Isoforms: Potential Specific Regulators of Neuronal and Cardiovascular Development
Source: PLoS One. 2015 Jun 17;10(6):e0129944. doi: 10.1371/journal.pone.0129944 (PMC4470590; doi:10.1371/journal.pone.0129944)

S1 Fig.

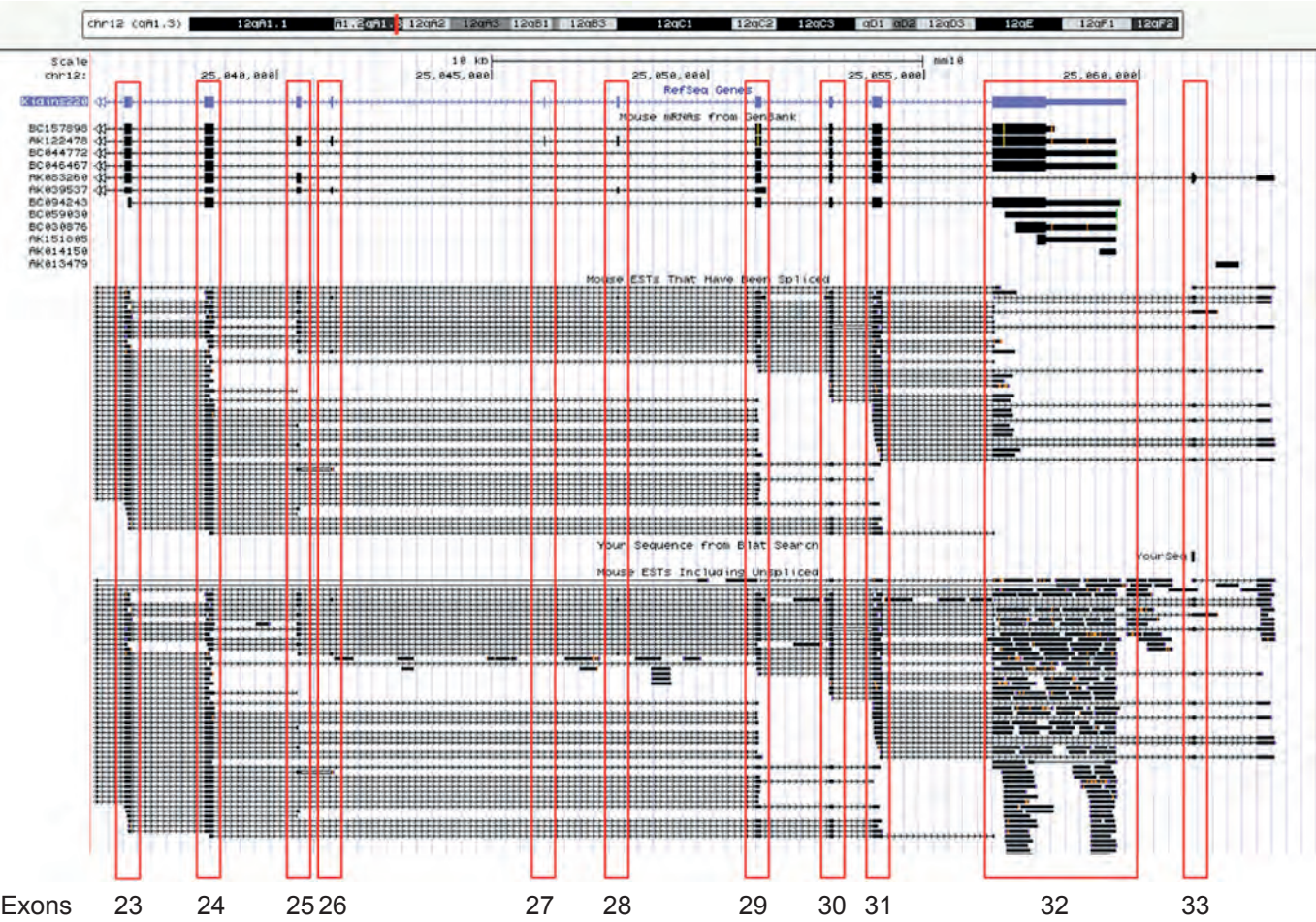

Supplement: S1 Fig — Schematic showing mouse Kidins220 exon-intron structure between exons 23 and 33 (NM_001081378) (www.genome.ucsc.edu). Horizontal lines represent different mouse ESTs. Numbered red boxes mark Kidins220 exons. (PDF) [file pone.0129944.s003.pdf]

S2 Fig.

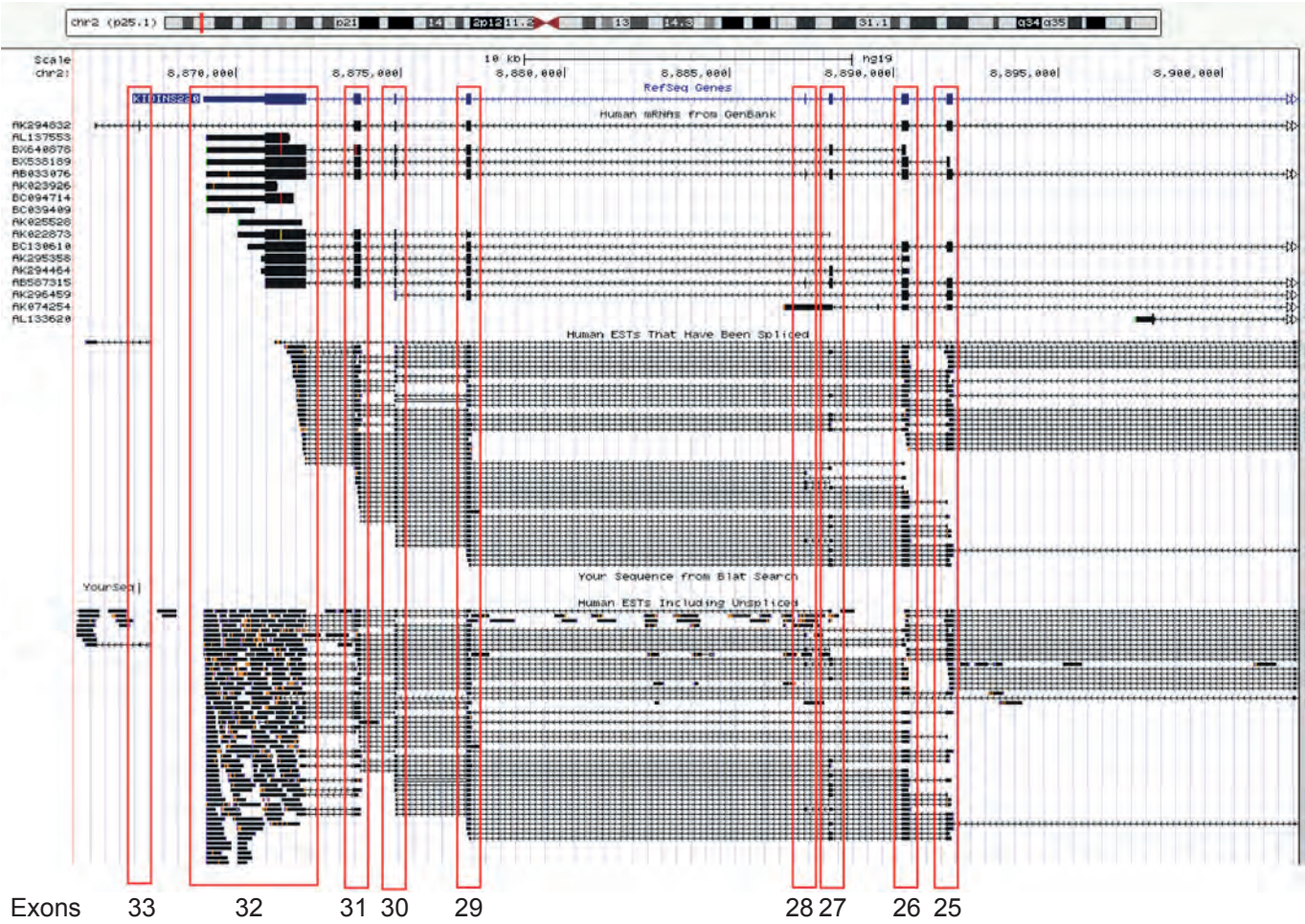

Supplement: S2 Fig — Schematic showing human Kidins220 exon-intron structure between exons 25 and 33 (www.genome.ucsc.edu). Horizontal lines represent different mouse ESTs. Numbered red boxes mark Kidins220 exons. (PDF) [file pone.0129944.s004.pdf]

S3 Fig.

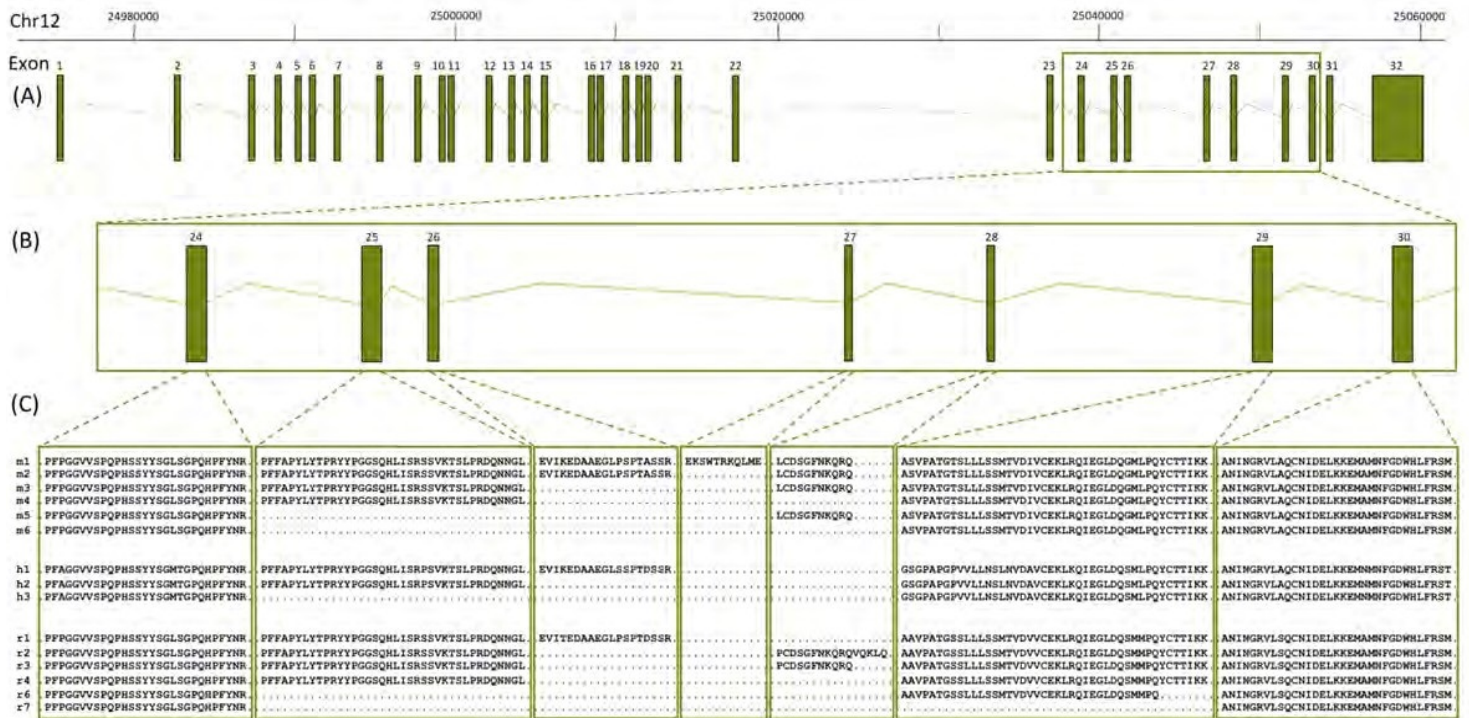

Supplement: S3 Fig — Schematic showing: (A) mouse Kidins220 (NM_001081378) exon-intron structure on chromosome 12 (Chr12), (B) area of interest between exons 24 and 30 and (C) sequence alignment of the mouse (m1-6), human (h1-3) and rat (r1-4, 6, 7) splice variants identified in this study and during the cloning of rat Kidins220 [34]. Vertical green rectangles and numbering refer to mouse exons. (PDF) [file pone.0129944.s005.pdf]

S4 Fig.

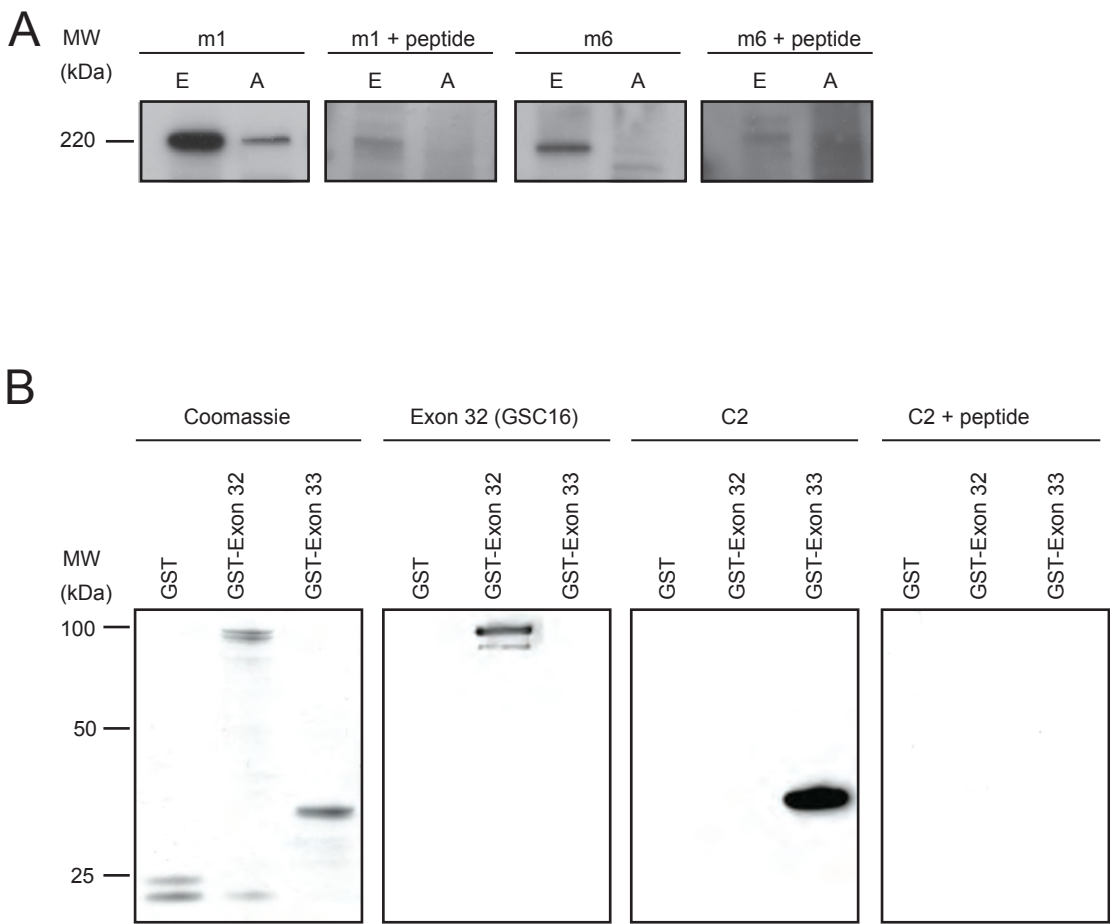

Supplement: S4 Fig — (A) Antibodies targeting the alternative splice isoforms m1 and m6 of mouse Kidins220 were tested by peptide competition in embryonic day 18.5 (E) and adult (A) brain lysates. Western blots were probed with the indicated antibodies with or without pre-incubation with the specific peptides (100 μM). (B) Recombinant GST fusion proteins of exon 32 and exon 33, together with control GST, were resolved in SDS-PAGE and either stained with Coomassie Blue R (left panel) or transferred on nitrocellulose and probed with the indicated antibodies (right panels). The rabbit polyclonal antibody GSC16 [31] recognised GST-exon 32, but not GST-exon 33, whereas the rabbit polyclonal antibody C2 specifically stained GST-exon 33. This signal was abolished by pre-incubating this antibody with an excess of the immunising peptide (100 μM; C2+peptide). (PDF) [file pone.0129944.s006.pdf]

S5 Fig.

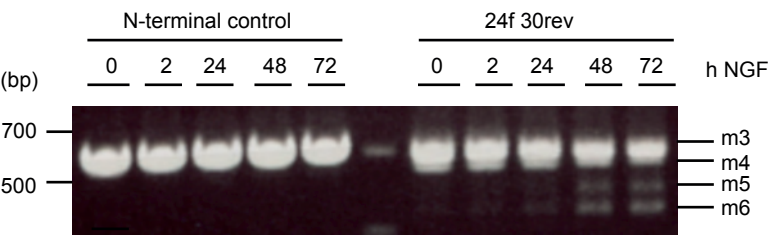

Supplement: S5 Fig — PC12 cells were maintained in control medium or treated with 100 ng/ml NGF for the indicated times. RNA was then extracted and reverse transcribed. N indicates PCR products obtained using primers designed to recognise exons 3 and 8. 24f-30r indicates samples obtained by amplification with primers recognising exons 24 and 30. (PDF) [file pone.0129944.s007.pdf]

S6 Fig.

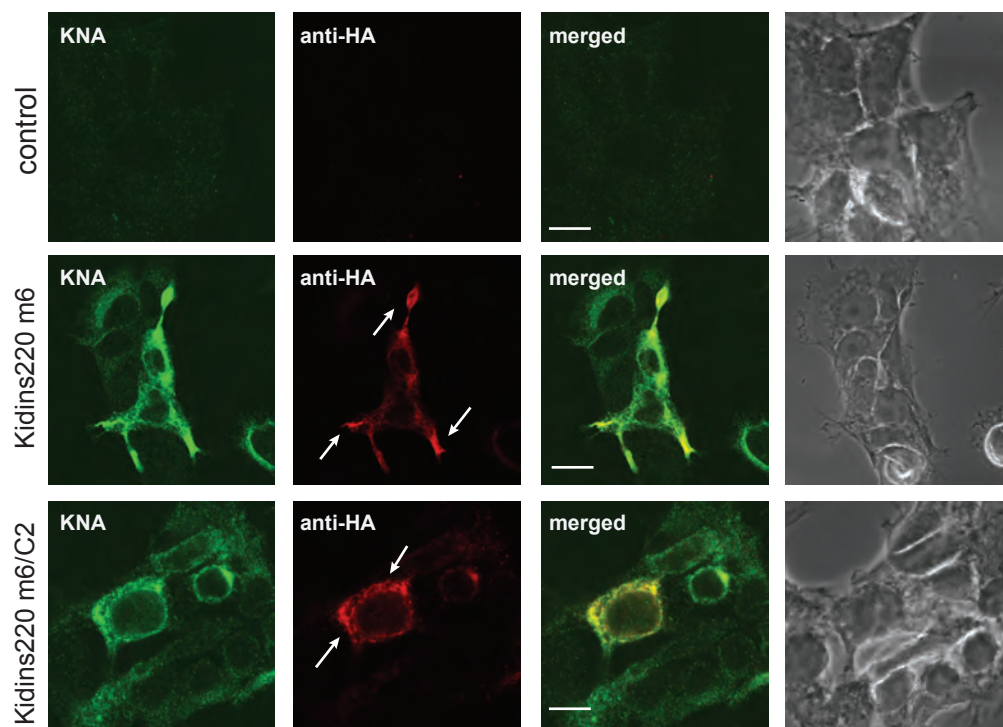

Supplement: S6 Fig — HEK cells were transfected with Tet-ON pLVX vector only (control), HA-tagged Kidins220 isoform m6 or isoform m6/C2 and stimulated with doxycycline for 24 h. The localisation of total Kidins220 was revealed using a polyclonal antibody directed against the amino-terminus (KNA antibody; in green). An anti-HA antibody was used to stain Kidins220 isoforms m6 and m6/C2 (in red). Arrows indicate distinct Kidins220 isoform patterns for isoforms m6 (plasma membrane and outgrowths) and m6/C2 (puncta in the cell body). Scale bars are 10 μm for the control and the Kidins220 m6/C2 panels, and 20 μm for the Kidins220 m6 panels. (PDF) [file pone.0129944.s008.pdf]

S7 Fig.

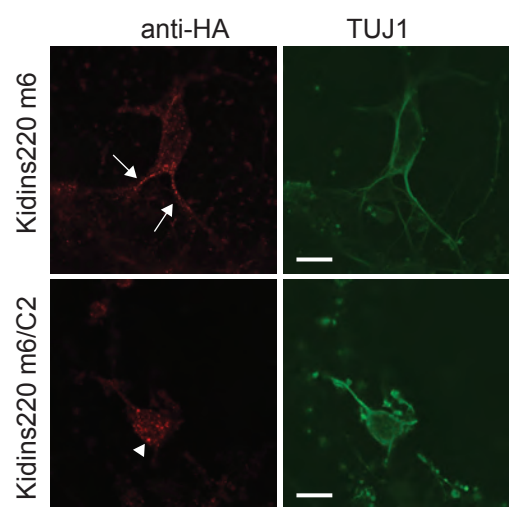

Supplement: S7 Fig — Hippocampal neurons were transfected with a Tet-ON pLVX vector encoding HA-tagged Kidins220 isoform m6 or isoform m6/C2 and after 4 h stimulated with doxycycline. An anti-HA antibody was used to stain Kidins220 isoforms m6 and m6/C2 (in red) after 48 h. The neuronal cytoskeleton was stained with TUJ1 (in green). Arrows indicate the presence of Kidins220 isoforms m6 in neurites. The arrowhead points to the somatic accumulation of the m6/C2 splice variant. Scale bars, 10 μm. (PDF) [file pone.0129944.s009.pdf]

S8 Fig.

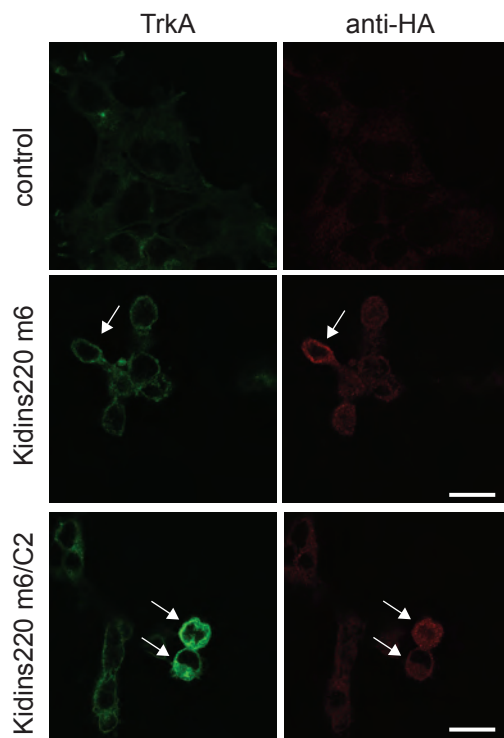

Supplement: S8 Fig — HEK-TrkA cells were transfected with a Tet-ON pLVX vector encoding HA-tagged Kidins220 isoform m6 or isoform m6/C2. Samples were stained for TrkA (in green) and for HA Kidins220 (in red). Arrowheads indicate transfected cells. All channels were adjusted equally to accommodate the increase in TrkA levels upon m6/C2 expression. Scale bars, 20 μm. (PDF) [file pone.0129944.s010.pdf]
